# The DANIsh VASculitis cohort study: protocol for a national multicenter prospective study including incident and prevalent patients with giant cell arteritis and polymyalgia rheumatica

**DOI:** 10.3389/fmed.2024.1415076

**Published:** 2024-07-03

**Authors:** Berit D. Nielsen, Salome Kristensen, Agnete Donskov, Lene Terslev, Lene Wohlfahrt Dreyer, Ada Colic, Merete Lund Hetland, Pil Højgaard, Torkell Ellingsen, Ellen-Margrethe Hauge, Stavros Chrysidis, Kresten K. Keller

**Affiliations:** ^1^Department of Medicine, The Regional Hospital in Horsens, Horsens, Denmark; ^2^Department of Rheumatology, Aarhus University Hospital, Aarhus, Denmark; ^3^Department of Clinical Medicine, Aarhus University, Aarhus, Denmark; ^4^Center of Rheumatic Research Aalborg (CERRA), Department of Rheumatology, Aalborg University Hospital, Aalborg, Denmark; ^5^Department of Clinical Medicine, Aalborg University, Aalborg, Denmark; ^6^DANBIO and Copenhagen Center for Arthritis Research (COPECARE), Center for Rheumatology and Spine Diseases, Centre for Head and Orthopedics, Rigshospitalet, Glostrup, Denmark; ^7^Department of Clinical Medicine, Faculty of Health and Medical Sciences, University of Copenhagen, Copenhagen, Denmark; ^8^Department of Rheumatology, Zealand University Hospital, Køge, Denmark; ^9^Department of Medicine (2), Holbæk Hospital, Holbæk, Denmark; ^10^Department of Rheumatology, Odense University Hospital, Odense, Denmark; ^11^Department of Rheumatology, University Hospital of Southern Denmark, Esbjerg, Denmark

**Keywords:** giant cell arteritis, polymyalgia rheumatica, imaging, prognosis, cohort study (or longitudinal study), prospective observational study

## Abstract

The DANIsh VASculitis cohort study, DANIVAS, is an observational national multicenter study with the overall aim to prospectively collect protocolized clinical data and biobank material from patients with polymyalgia rheumatica (PMR) and giant cell arteritis (GCA) diagnosed and/or followed at Danish rheumatology departments. A long-term key objective is to investigate whether the use of new clinically implemented diagnostic imaging modalities facilitates disease stratification in the GCA-PMR disease spectrum. In particular, we aim to evaluate treatment requirements in GCA patients with and without large-vessel involvement, treatment needs in PMR patients with and without subclinical giant cell arteritis, and the prognostic role of imaging with respect to aneurysm development. Hence, in GCA and PMR, imaging stratification is hypothesized to be able to guide management strategies. With an established infrastructure within rheumatology for clinical studies in Denmark, the infrastructure of the Danish Rheumatologic Biobank, and the possibility to cross-link data with valid nationwide registries, the DANIVAS project holds an exceptional possibility to collect comprehensive real-world data on diagnosis, disease severity, disease duration, treatment effect, complications, and adverse events. In this paper, we present the research protocol for the DANIVAS study.

**Clinical trial registration**: https://clinicaltrials.gov/, identifier NCT05935709.

## Introduction

In recent years, research advancements in giant cell arteritis (GCA) and polymyalgia rheumatica (PMR) have improved diagnosis and treatment approaches. Imaging tests have become integral in diagnosing GCA, improving diagnostic reliability, promoting fast diagnosis, and expediting treatment initiation, thereby reducing complications (1–10). Vascular ultrasound has high diagnostic accuracy, is a cheap and non-invasive procedure, and can be performed bedside. Therefore, vascular ultrasound of temporal and axillary arteries is recommended as a first-line diagnostic tool in patients suspected of GCA. A whole-body 18F FDG PET/CT and a cranial MR have comparable diagnostic properties and can be used as alternatives or in unresolved patients (9, 10). Few studies exploring the potential value of imaging in PMR have been performed (11–15), but recent reports suggest an overall benefit of early referral and specialist care of PMR patients (16–20). Additionally, new glucocorticoid-sparing treatment options for GCA and PMR have emerged, and several clinical trials are ongoing (18, 21).

Despite these improvements in GCA and PMR management, several unmet needs call for systematic prospective observational and long-term follow-up studies in real-world settings.

GCA and PMR have different initial glucocorticoid requirements, but a long-term glucocorticoid -tapering regime over 1–2 years is the treatment target for both diseases (6, 22, 23). However, relapses are frequent, and longer treatment is often required carrying a high risk of glucocorticoid adverse events (24–29). In addition, the risk of complications and the lack of valid clinical tools to assess activity imply a high risk of over-treatment. Specific indications for initiation and optimal timing of tapering or discontinuation of IL-6 inhibitor treatment remain unresolved (30–37). To select patients who gain the most from early add-on steroid sparring therapy and to guide treatment strategy, baseline stratification tools and disease activity biomarkers are highly needed.

Despite the diagnostic value of imaging and its sensitivity to change after the institution of glucocorticoids, its prognostic value and ability to discriminate remission and relapse in clinical routine care remain less clear (7, 9, 25, 38–41). Imaging facilitates new insight into disease distribution and severity that may have prognostic potential, as, for example, discriminating large-vessel and cranial vessel involvement in GCA or by identification of subclinical GCA in phenotypic PMR (42).

Epidemiologic studies and smaller cohort studies consistently report an increased risk of vascular complications such as aortic aneurysms and dissection later in the disease course of GCA (43–46). Although the development of aortic aneurysm and dissection can be fatal, incidence rates are still low and progression rates vary (46). Current guidelines only recommend screening for aortic complications on an individual basis but do not provide any guidance for the identification of patients at risk (6, 10, 47).

In Denmark, the optimal conditions for the establishment of a national GCA and PMR research collaboration exist. The highest incidences of GCA and PMR are found in the Scandinavian countries (24), and all GCA patients and many PMR patients are evaluated and diagnosed by a rheumatologist. In addition, within the Danish Rheumatology Society, established experience and infrastructure for clinical cohort studies in GCA and PMR and the Danish Rheumatologic Biobank are present (2, 8, 48, 49). In line with Danish and European guidelines, imaging has been gradually implemented, allowing imaging-based disease characterization. Furthermore, linkage to Danish nationwide administrative registries with data on, e.g., diagnosis, prescriptions, laboratory and pathology results, time, and cause of death is available and provide important complementary data (50). Alignment with similar European cohorts has been strived for when selecting data variables for the study and developing the DANIVAS data collection instrument. Taken together, the DANIVAS cohort study will include crucial data providing new insight into GCA/PMR management and disease course, with a particular emphasis on the prognostic value of imaging-based disease stratification. Even more, DANIVAS enables data comparison across cohorts and supports future international research collaboration.

In this paper, we present the protocol for the DANIVAS study.

### Study design

A national multicenter prospective observational study of incident and prevalent patients with PMR and GCA diagnosed and/or followed at Danish rheumatology departments.

Descriptive clinical data are collected in a web-based, clinician-driven database, and blood samples are collected through the infrastructure of the Danish Rheumatologic Biobank. Complementary data are obtained from national administrative registries.

### Study objectives

The overall aim of the DANIVAS cohort study is to improve disease control and reduce disease- and treatment-related damage in GCA and PMR. The study objective is to investigate the use of new diagnostic imaging modalities for facilitating disease stratification that can potentially predict treatment requirements and complications and hence guide management strategies. Specific primary, key secondary, secondary, and exploratory objectives are listed in Table 1.

**Table 1:** Catalog of study objectives.

**Primary objective**	
1	To compare cumulative GC doses in patients with isolated c-GCA as compared to LV-GCA (with or without c-GCA).
**Key secondary objectives**	
1	To compare cumulative GC doses in patients with pure PMR* compared to PMR patients with subclinical LV-GCA.
2	To compare the incidence of aortic dilatation 2 years after diagnosis in patients with c-GCA as compared to LV-GCA (with or without c-GCA).
3	In the subpopulation of patients in whom a diagnostic FDG PET/CT was performed at diagnosis, to evaluate the risk of aortic complications (aneurysms and dissections) in GCA patients with aortic involvement as compared to patients without aortic involvement.
**Secondary objectives**	
1	To compare treatment response, risk of relapse, need for GC-sparring add-on treatment, and disease duration in patients with c-GCA as compared to LV-GCA (with or without c-GCA).
2	To compare treatment response, risk of relapse, need for GC-sparring add-on treatment, and disease duration in patients with pure PMR compared to PMR patients with subclinical LV-GCA.
3	In the subpopulation of patients in whom a diagnostic FDG PET/CT was performed, to evaluate the association between aortic FDG uptake and aortic dilatation at year 2.
**Exploratory objectives**	
1	To identify clinical features associated with the different disease subsets, c-GCA, LV-GCA, and PMR.
2	To assess and evaluate risk factors and biomarkers predicting GCA in patients with PMR.
3	To assess and evaluate incidence, prevalence, and predictors of ischemic events and vascular complications in GCA patients.
4	To assess and evaluate incidence, prevalence, and predictors of comorbidity in GCA and PMR patients.
5	To assess and evaluate diagnostic strategies and implementation of diagnostic imaging in GCA and PMR.
6	To evaluate adherence to clinical guidelines.
7	To evaluate and predict treatment efficacy, safety, and predictors of treatment success, treatment failure, and maintenance of remission after therapy withdrawal.
8	To assess and evaluate risk factors and biomarkers predicting vascular complications in GCA.

*Pure PMR; PMR patient without cranial GCA symptoms, new-onset claudication, or GCA. c-GCA, cranial giant cell arteritis; LV-GCA, large-vessel GCA; PMR, polymyalgia rheumatica; GC, glucocorticoid; ^18^F-FDG PET/CT, fluorine-18-fluorodeoxyglucose positron emission tomography with computed tomography.

On top of the specific research objectives, the systematic collection of prospective clinical data and biobank blood samples provides a fundamental basis for future research projects and a scientific framework for Danish GCA/PMR researchers and for international research collaborations.

### Methods and analysis

#### Data collection and setting

Clinical data (including imaging, blood tests, and histology), demographics, patient-reported outcomes, and biobank samples will be collected at baseline and during follow-up for as long as patients are seen in the rheumatology departments. For an angiographic sub-study, structural damage of the aorta will be assessed in a subset of GCA patients 2 years after diagnosis. Data on long-term complications, comorbidity, death and migration, and time and amount of retrieved glucocorticoid prescriptions will be collected through Danish registries.

Patients will be treated according to the Danish national treatment guideline for GCA and PMR, which adhere to current European recommendations (6, 22, 23).

The study was registered in ClinicalTrials.gov (NCT05935709) on 28 June 2023.

#### Recruitment

Patients will be recruited from Danish rheumatology departments at routine visits either at the time of diagnosis or during the disease course.

Enrollment was initiated on 1 November 2023 from two centers in order to test the feasibility of study organization and data collection. Within the next year, all rheumatology departments at Danish hospitals will be invited to participate in the study. The last patient’s first visit is expected by the end of 2039.

The potential for recruitment from Danish rheumatology departments is excellent. High referral rates can be expected according to the Danish national GCA and PMR management guidelines that encourage PMR evaluation by rheumatologists and recommend that all patients suspected of GCA are referred for prompt diagnostic evaluation by a rheumatologist, the latter including diagnostic imaging performed in a hospital setting.

#### Study population

##### Sample size

In-depth disease characterization by the time of inclusion, including reporting on vessel involvement according to imaging results, allows for both incident and prevalent GCA and PMR patients to contribute to the primary and secondary outcomes. A sample size calculation was made based on the primary outcome reaching a total number of 3,000 GCA patients to be included. Assuming a pooled standard deviation of 8,500 mg (24, 51), and an equal distribution between the groups (c-GCA and LV-GCA), each group requires 1,519 samples to achieve 90% power and a 5% significance level (two-sided) for detecting a true difference in mean cumulated glucocorticoid dose of 1,000 mg between the two groups. Based on incidence rates and the proportion of referrals, we expect to be able to include the same number of PMR patients (16, 18). Expecting subclinical GCA in 20% of PMR patients and assuming a similar pooled standard deviation (52, 53), 1,370 PMR patients are needed to achieve 90% power and a 5% significance level (two-sided) for detecting a true difference in mean cumulated glucocorticoid dose of 2000 mg between the two groups (PMR with and without subclinical GCA).

##### Eligibility

Patients can be included at any time during the disease course. By the time of inclusion, patients will be registered as either incident (newly diagnosed within the last 3 months) or prevalent (included during routine follow-up >3 months after and ≤ 5 years after diagnosis) cases. Inclusion criteria are as follows:

- GCA and/or PMR diagnosis established or confirmed by a rheumatologist (clinical expert opinion), and
- Speak and understand Danish, and
- Are able to give signed and dated informed consent.

Patients diagnosed with other systemic autoimmune diseases that out-rule the diagnosis of GCA or PMR and patients diagnosed >5 years ago will not be included.

For participation in the angiography sub-study, patients included more than 2 years after diagnosis or with contraindications for the angiography (claustrophobia, body weight > 150 kg, pacemaker, metallic foreign body, and eGFR<30) will not be included.

#### Visits

**Figure 1:**
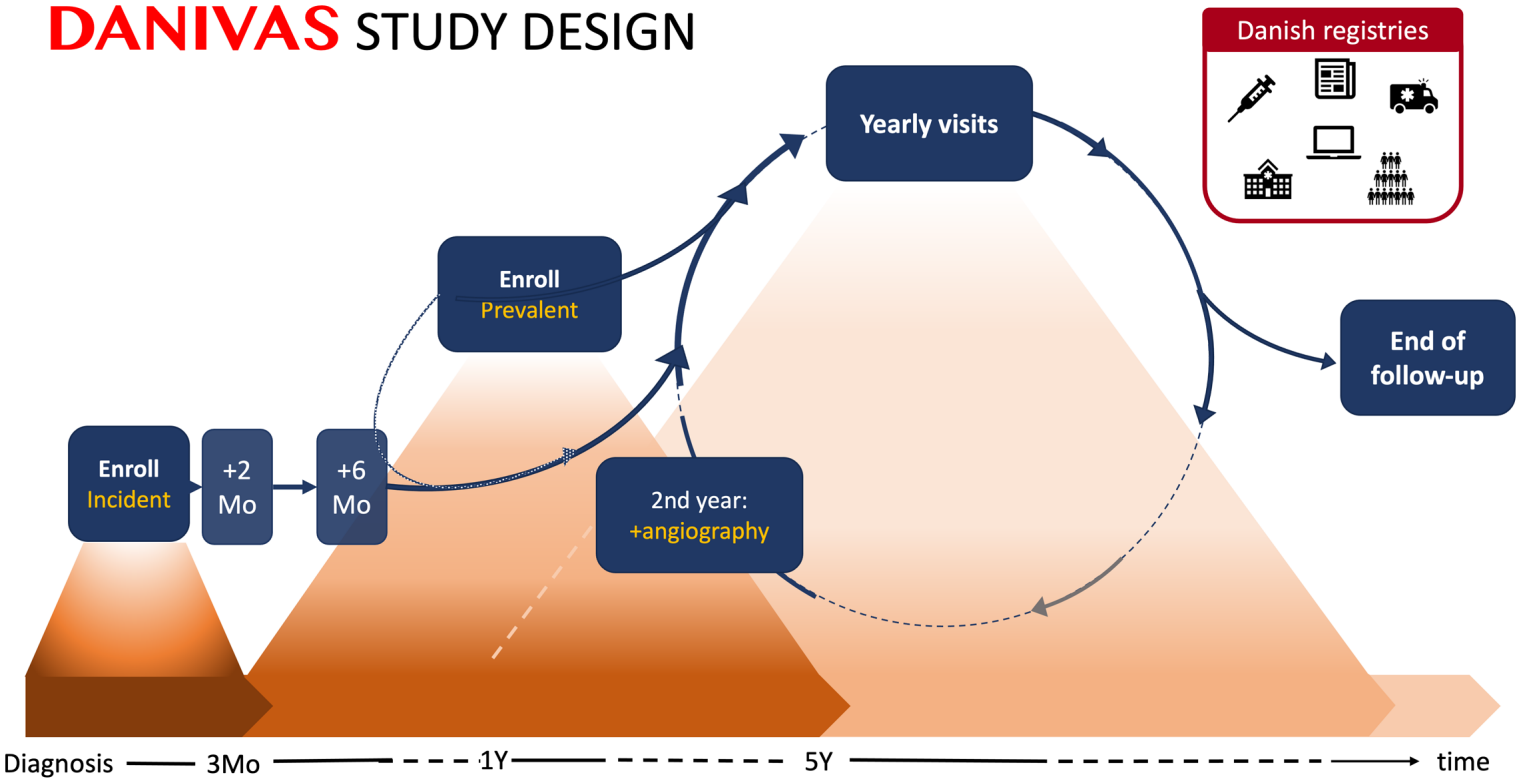
DANIVAS study design. Patients are enrolled at any time during their disease course (≤5 years disease duration) and will be registered as either incident (within 3 months of diagnosis) or prevalent. Study visits will be performed 2 (response visit) and 6 months after diagnosis (incident patients and prevalent patients included <4 months after diagnosis) and subsequently every year (all patients). At the 2-year follow-up, aortic angiography will be performed in a subset of GCA patients. Data collection for the enrollment visits and other study visits (2-month response visit, 6 months visit, and annual visits) are described in more detail in the ‘*Data collection’* section and Table 2. Clinical follow-up is terminated by the time of stable drug-free remission or dismission from rheumatology care, on patient’s request or in the event of death, migration, non-compliance, or if the diagnosis is dismissed. Linkage of data across nationwide medical and administrative registries at the individual level will be performed to enrich outcome and covariate data. Mo, month; Y, year.

The study visit schedule is adapted to standard programs for managing PMR and GCA patients in routine clinical care. In routine rheumatology care in Denmark, GCA patients are typically followed up 2–12 months after treatment discontinuation, while the follow-up schedule for PMR patients varies. The study design is illustrated in Figure 1. The following study visits will be conducted:

- Enrollment visit: First visit to obtain informed consent and collect master data regarding diagnostic subgroup classification and demography.
- Response visit: Second visit 2 months after diagnosis (only incident patients).
- Routine study visits: Six months after diagnosis (incident patients and prevalent patients included <4 months after diagnosis) and subsequently every year as long as patients are seen at the rheumatology department.
- Aortic screening visit: Two years after diagnosis, screening for aortic complications will be performed
- Withdrawal visit: Visit to complete study participation due to stable drug-free remission or dismission from rheumatology care, patients’ request, or in the event of death, migration, non-compliance, or if GCA/PMR diagnosis is dismissed.

#### Data collection

An overview of the data collection at each study visit is presented in Table 2. All procedures, but the 2-year angiography, are performed on clinical indication according to clinical guidelines as part of routine care. As not all routine care visits are necessarily performed as study visits, the data collection at each study visit also serves the purpose of summarizing disease-related medical events in the interim period.

**Table 2:** Schedule of procedures and assessments at baseline, follow-up, and withdrawal.

Visit*		Enrollment visit	Response visit	Routine visit	Aortic screening visit	Withdrawal visit
**Study procedures**	**Time of visit (time from diagnosis)**	Diagnosis	2 Mo	6th and every 12 Mo	2 Y	
	**Margin**	+ 5 years	+/−1 month	+/− 2 months	+/− 6 months	
	Attendance data	X	X	X		X
	Eligibility and consent	X				
	Demography	X				
	Diagnostic characteristics, including disease stratification	X	(X)	(X)	X	X
	Clinical evaluation		X	X	X	X
	Medicine and adverse events		X	X	X	X
	Complications		X	X	X	X
	Comorbidity		X	X	X	X
	PROMs		X	X	X	X
	Laboratory tests including biobank		X	X	X	X
	Angiography of aorta				X	
	Reason for withdrawal					X
	**Optional §**					
	Musculoskeletal ultrasound	X	X	X		
	Vascular ultrasound	X	X	X		
	[^18^F] FDG PET/CT	X	X	X		
	TAB	X				

*Type of visit (enrollment, response, routine study, aortic screening, and withdrawal) is described in the section ‘Visits’. An enrollment visit is always performed together with a response or routine visit. Response visit is performed in incident patients after treatment initiation. Routine study visits will be performed as long as patients are followed in the rheumatology outpatient clinic. Withdrawal visit procedures will be performed in all cases where it is possible. Reason for withdrawal will always be obtained. §Imaging and/or TAB is performed at the physician’s discretion and on clinical indication. The type of imaging, e.g., diagnosis may vary depending on the patient and the study site. MO, months; Y, years; PROMs, patient-reported outcome measures; [^18^F] FDG PET/CT, ^18^F-FDG PET/CT, fluorine-18-fluorodeoxyglucose positron emission tomography with computed; TAB, temporal artery biopsy.

##### Diagnostic information and referral history

Diagnostic information regarding symptom onset, presentation, referral history, diagnostic work-up, time and type of clinical diagnosis (GCA and/or PMR as considered by the treating physician), and initial treatment will be recorded by the time of enrollment.

For patients with a clinical diagnosis of GCA, it will be recorded if a diagnostic test, that is temporal artery biopsy, vascular ultrasonography, 18F FDG PET/CT, MR, or CT-angiography, was conducted or not, and if so, whether the result was positive, inconclusive, or negative for GCA diagnosis.

For patients with a clinical diagnosis of PMR, it will be recorded if the following diagnostic tests either supporting PMR or excluding differential diagnosis was performed: musculoskeletal hip and shoulder ultrasonography, 18F FDG PET/CT, negative vascular ultrasonography, computed tomography of chest, abdomen, and pelvis, or other investigation to evaluate potential malignancy. Assessment of the variables included in the 2022 ACR/EULAR classification criteria for GCA and/or 2012 EULAR classification criteria for PMR will be registered.

##### Definition of imaging-based disease stratification groups

The Danish Society of Rheumatology endorses adherence to EULAR recommendations regarding the diagnostic evaluation of patients suspected of GCA and/or PMR (6, 10, 23). Consequently, we expect GCA patients to have at least one vascular imaging procedure performed, and in case of negative or inconclusive results. That additional tests to confirm diagnosis will be made. Vascular ultrasonography, including the assessment of temporal and axillary arteries as a minimum, is performed by trained rheumatologists, and recommended technical and procedural requirements are met (10, 54). Diagnostic conclusions are made according to OMERACT definitions (55). Recording of other imaging and pathology results relies on the radiology/pathology report and is interpreted according to procedural recommendations and accepted diagnostic criteria (56, 57).

GCA patients with a positive imaging test or histology will be categorized according to vessel involvement as ‘c-GCA’ and/or ‘LV-GCA’. C-GCA is defined as the involvement of cranial arteries including, but not limited to temporal, facial, occipital, maxillary, and vertebral arteries, whereas LV-GCA is defined as the involvement of extracranial large arteries including but not limited to aorta and/or its primary branches (e.g., carotid, subclavian, axillary, and femoral arteries). If applicable, the presence or absence of aortitis will be recorded.

In patients with concomitant GCA and PMR, it will be recorded if GCA is subclinical, that is vasculitis is diagnosed by imaging or histology in the absence of cranial or claudication symptoms attributed to GCA (12).

Diagnosis and disease stratification will continuously be revised according to diagnostic test results available (at study visits and retrospectively).

##### Demography

Age, gender, height, weight, and history of smoking and alcohol consumption will be recorded at enrollment.

##### Clinical evaluation

New or persistent symptoms and findings of GCA and (58) will be recorded at each visit if present. Cranial symptoms and findings recorded include headache, scalp tenderness, jaw or tongue claudication, visual disturbances (sight loss, amaurosis fugax, and double vision), abnormal temporal artery (tender, swollen, and pulseless), scalp necrosis, transient ischemic attack, or stroke. Large-vessel symptoms include arm or leg claudication, carotidynia, brachial blood pressure difference > 10 mmHg, pulselessness, or large-vessel bruits. PMR symptoms and findings recorded include symmetric shoulder pain and stiffness, symmetric hip pain and stiffness, mobility of upper arms, PMR activity score (58), RS3PE (remitting seronegative symmetrical synovitis with pitting edema), and peripheral arthritis. Constitutional symptoms include fever, weight loss, night sweats, and malaise. New symptoms are defined as new onset or worsening within 4 weeks, whereas symptoms lasting without worsening for >4 weeks are considered persistent. The physician’s assessment of disease activity based on clinical evaluation (physician NRS and physician disease activity category; remission, potential relapse without treatment escalation, relapse (treatment escalation), and refractory disease) will be recorded. Relapses will be categorized as minor or major according to EULAR definitions (10). Any relapse that leads to treatment intensification since the last study visit will also be recorded.

##### Medical history and adverse events

At each visit, the current dose of glucocorticoid, tsDMARD, and/or bDMARD treatment and changes since the last visit are recorded. Date of start, change, and discontinuation of immunosuppressive therapy and reasons (start or increase: risk of disease complications, refractory disease, repeated relapses, relapse on unacceptable high glucocorticoid doses, adverse effects from other immunosuppressive therapy, and comorbidity. Discontinuation or decrease: remission, adverse events, or no effect) for these will be recorded.

##### Complications

At each visit, potential disease-related complications, including visual impairment and vascular complications including aortic dilatation and aortic dissection, and the time of the event will be recorded.

##### Comorbidity

Comorbidity including cardiovascular disease, hypertension, hypercholesterolemia, diabetes, chronic kidney disease, osteoporosis, chronic lung disease, infections, or malignancies will be recorded at the baseline visit. Routine clinical monitoring of HbA1c levels and results of DXA scans will be recorded continuously.

##### Patient-reported outcome measures (PROMs)

At each visit, patients will be asked to report their global disease activity [Numerical Rating Scale (NRS), 0–10] and the duration of morning stiffness (minutes) will be recorded.

##### Laboratory tests and biobank

Routine blood analysis, including C-reactive protein and glycated hemoglobin (HbA1c), will be performed as a standard of care. Additionally, biobank blood samples for future research purposes are collected by the infrastructure of the clinical biobank *Danish Rheumatologic Biobank* under the interregional Bio- and Genome Bank Denmark. Biobank blood samples will be collected at each study visit.

##### Aortic screening visit

In a subpopulation of GCA patients, an aortic angiography will be scheduled 2 years after diagnosis to screen for aortic dilatation and aneurysms. The angiography can be performed as either CT or MR angiography and will be performed according to local set-up and imaging acquisition protocols. Subsequent aortic imaging will be performed on clinical indications at an individual basis at the discretion of the treating physician.

## Linkage with registries

Danish residents receive a unique 10-digit civil registration number by the time of birth or immigration that ensures the linkage of data across nationwide health and administrative registries at the individual level (Table 3). This linkage will ensure the collection of data on events, and treatment occurring after consultations in rheumatology departments has been terminated as well as the follow-up time.

**Table 3:** Data from national registries.

**Outcomes**	**Type of data**	**Registry**	**Time period**
**Primary outcome**	Redeemed prednisolone prescriptions including time of redemption, dosage, number of packages, number of tablets	Danish National Prescription Registry	From index date* to death, emigration, or end of follow-up
**Secondary outcomes**	Aortic aneurysm, dissections, peripheral artery disease, aortic surgery, amputation	Danish National Patient Registry (DNPR)	From 5 years before the index date to death, emigration, or end of follow-up
		The Danish Cause of Death Registry	Index date to death
**Exploratory**	Vascular complications: Aortic aneurysm, dissections, peripheral artery disease, aortic surgery, amputation, blindness, low vision, visual disturbances, acute myocardial infarction, ischemic stroke, percutaneous coronary intervention (PCI), and coronary artery bypass grafting	Danish National Patient Registry (DNPR)	From 5 years before the index date to death, emigration, or end of follow-up
		The Danish Cause of Death Registry	Index date to death
	Safety: Osteoporosis and osteoporotic events, infections, hypertension, myopathy, adrenal insufficiency, psychosis, gastrointestinal perforation, peptic ulcer, avascular necrosis, cataract, glaucoma	Danish National Patient Registry (DNPR)	From 5 years before index date* to death, emigration, or end of follow-up**
	Cause of death	The Danish Cause of Death Registry	Index date to death
**Covariates**	Diagnoses contained in the Charlson Comorbidity Index**	Danish National Patient Registry (DNPR)	From 5 years before the index date to death, emigration, or end of follow-up
	HbA1c and cholesterol (total, LDL, HDL, and triglycerides)	The Registry of Laboratory Results for Research	From 5 years before the index date to death, emigration, or end of follow-up
**Follow-up time estimation**	Death and emigration	Danish Civil Registration System	From index date to end of follow-up

* Index date; Date of diagnosis. **The exploratory outcomes will be evaluated after 5 years and by the end of follow-up. **Charlson comorbidity index (59): Myocardial infarction, congestive heart disease, vascular disease, cerebrovascular disease, dementia, chronic pulmonary disease, connective tissue disease, ulcer disease, liver disease, diabetes, hemiplegia, renal disease, tumor (+/−metastatic) leukemia, lymphoma, AIDS.

The cumulated dose of glucocorticoid, time of glucocorticoid treatment, and potential treatment-free remission will be estimated based on redeemed prednisolone prescriptions obtained through the Danish National Prescription Registry (DPR).

Linkage with the Danish National Patient Registry (DNPR), the Danish Cause of Death Registry, The Registry of Laboratory Results for Research (LABKA), and the Danish Civil Registration System is performed to enrich data regarding vascular complications, treatment-related complications, potential confounding diseases, time to event, death, immigration, or censoring.

### Outcomes and data analysis plan

#### Primary outcome

1. Cumulative glucocorticoid doses will be calculated based on redeemed prescriptions from the time of diagnosis to the end of follow-up (date of data extraction, death, or emigration). The difference between LV-GCA (with and without c-GCA) and isolated c-GCA, as characterized in the DANIVAS database will be compared by Student’s t-test or Wilcoxon Mann–Whitney U-test.

#### Key secondary outcomes

1. Cumulative glucocorticoid doses will be calculated based on redeemed prescriptions from the time of diagnosis to the end of follow-up (date of data extraction, death, or emigration). The difference between patients with pure PMR compared to PMR patients with subclinical LV-GCA will be compared by Student’s t-test or Wilcoxon Mann–Whitney U-test.
2. The incidence of aortic dilatation 2 years after diagnosis in patients with isolated c-GCA as compared to LV-GCA (with or without c-GCA) will be compared by chi-square test. Incidences will be calculated as proportions, that is events per patient at risk, and as incidence rates, that is number of events divided by the sum of the person-time of the at-risk population. Associated 95% binomial confidence intervals will be calculated.
3. The risk of aortic complications (aneurysms and dissections) in GCA patients with aortic involvement as compared to patients without aortic involvement (in the subpopulation of patients diagnosed by PET/CT) will be compared by chi-square test and the association between baseline aortic FDG uptake intensity and aortic diameter at year 2 will be evaluated by linear regression or Spearman correlation.

For the primary and key secondary outcome 2, subgroup analysis will be performed on patients diagnosed with PET/CT and ultrasound, respectively. In addition, an analysis comparing cumulative glucocorticoid doses in isolated LV-GCA as compared to patients with c-GCA (with or without LV-GCA) will be performed.

### Study organization, collaboration, and patient involvement

DANIVAS is led by a steering committee who has the overall scientific, organizational, and economic responsibility for DANIVAS. A DANIVAS research collaboration network of researchers within the field of GCA and PMR is built and will facilitate new research projects building upon the infrastructure of, and the teamwork within, DANIVAS.

Two patient research partners will be part of the steering committee. Patient research partners will be included in the research project according to” the European League Against Rheumatism recommendations for the inclusion of patient representatives in scientific projects” (60). In the selection of potential patient partners, communication skills, motivation, and constructive assertiveness in a team will be taken into account. Patient partners will prospectively ensure patients’ perspective on the relevance, feasibility, and added value of research initiatives as well as contribute to any needed adjustment of the study organization.

Through international research networks within the field of GCA and PMR, alignment of data collection with related European prospective GCA/PMR cohorts that are currently being developed was strived for in order to facilitate data comparison and future collaboration.

### Data collection and management

Data collection is documented in the individual electronic Case Report Form. To ensure high data completeness, the data manager at the Department of Rheumatology, Aarhus University Hospital, monitors data completeness and a built-in notifications system automatically sends alerts to the site investigators in case of missing visits and, if unsolved, ultimately to the data manager.

## Discussion

Although the implementation of diagnostic imaging has increased the awareness of the impact of disease extent and severity and led to the interpretation of GCA and PMR as overlapping diseases with a spectrum of disease manifestations and treatment requirements, the clinical impact on management and the therapeutic consequences remains mainly unsolved (42, 47).

Higher relapse rates or longer treatment needed for LV-GCA as compared to c-GCA has been reported in some studies (61–66), but not in all (67–70). In general, many of these studies are small, retrospective, and prone to selection or misclassification bias. Therefore, data to fully support early initiation of glucocorticoid-sparring therapy based on stratification by vessel involvement are still lacking.

Subclinical GCA occurs in approximately 20% of PMR patients (52, 53). However, many of these studies were performed in selected cohorts, questioning the true incidence. Nevertheless, a recent study reported higher relapse rates for PMR patients with subclinical GCA as compared to pure PMR patients (13) and smaller studies indicated a noteworthy risk of ischemic complications in this subgroup (71, 72). Accordingly, the routine diagnostic approach for patients with suspected PMR and the standard of care needed for patients with subclinical GCA need further evaluation (13).

Prospective long-term observational data of larger cohorts that allow for the evaluation of risk factors and prognostic biomarkers to identify patients at risk of aortic aneurysms and dissection and to reduce numbers needed to screen are lacking. Screening for aortic aneurysm would allow for timely surgical intervention to prevent aortic rupture. Although the relative risk for aortic rupture in GCA is high, the overall incidence rate is still low and time to event uncertain, challenging the development of screening algorithms (22, 46). Recent studies have indicated a positive association between the presence of vessel inflammation and subsequent vessel damage and a potential prognostic role of the presence of large-vessel involvement and the risk of aortic aneurysms (39, 45, 73, 74).

With systematic disease characterization including diagnostic imaging, which is highly implemented in the clinical care of GCA and PMR in Denmark, the DANIVAS cohort study provides essential data to address these needs.

Important differences between the results of real-world observational studies and RCT or single-center expert studies in GCA and PMR have been found and call for high-quality real-life data (2, 31, 75). Moreover, the potential drawbacks of smaller single-center cohort studies such as selection bias, lack of statistic power, and limited external validity can be overcome by a protocolized prospective national cohort study including GCA and PMR patients from both secondary and tertiary hospitals. The linkage of clinical data, including comprehensive disease characteristics, and registry data on an individual level provides unique insight into GCA and PMR disease courses. Hence, the DANIVAS cohort study holds the potential to improve diagnostic strategies and identify biomarkers of disease activity and prognosis, possibly providing tools to be implemented in daily clinical practice to personalize treatment strategies and hence improve effectiveness and safety. Moreover, translation and validation of the newly developed GCA-PRO and steroid-PRO to Danish versions are currently being performed. Incorporating these into DANIVAS in the future will gain supplementary information reflecting the impact of disease and its treatment on health-related quality of life (HRQoL) from the patient’s perspective (76, 77).

As a nationwide study aiming for inclusion and registration in everyday clinical care of patients with GCA and PMR in hospitality settings both with and without research experience within the field, the study comes with potential limitations. First, inclusion and data collection rely upon the clinician’s effort and may compete with other clinical obligations. Consequently, we cannot ensure all GCA and PMR patients are enrolled in the study. Careful pragmatic selection of data variables to be collected has been performed in order to ensure feasibility in a clinical context. However, this may also exclude appreciated but non-essential characteristics or confounders or outcomes. For instance, we did not find it possible to prioritize the collection of detailed vascular ultrasound data including the newly developed OGUS score or the complete set of variables included in the glucocorticoid toxicity index. For the latter, selected items can be obtained through the linkage with registries. Clinical data collection feasibility is currently being tested by clinicians, and data collection instruments and variables are adjusted if needed.

Although the majority of patients diagnosed with GCA are seen in hospital settings, and current guidelines endorse rheumatologic diagnostic evaluation of PMR patients also, not all patients with PMR are seen or followed in secondary care and the PMR cohort may be prone to selection bias toward more complicated cases.

Although imaging is recommended to establish GCA diagnosis and supplementary tests should be performed in patients with negative or inconclusive results, a smaller proportion of patients with a clinical diagnosis of GCA may not have a positive diagnostic test that allows stratification into defined disease subsets. Our primary outcome will be analyzed in patients that can be categorized based on diagnostic tests as described. A sensitivity analysis including patients with negative diagnostic tests will be performed stratifying these patients according to clinical symptoms.

Routine diagnostic imaging for GCA includes both cranial and large-vessel assessment. However, only evaluation of selected cranial and large vessels is needed to establish a diagnosis, potentially misclassifying some patients. However, it is well established that including axillary artery assessment, which is currently part of routine vascular ultrasonography examination in GCA, increases overall sensitivity (9) and also that axillary ultrasound depicts the majority of LV-GCA patients when PET/CT is used as a reference diagnosis(8). Screening for subclinical GCA in PMR is a matter of debate and may not need to be assessed in all PMR patients. However, diagnostic imaging is increasingly implemented in Denmark and cranial and large-vessel diagnostic imaging is performed in the majority of GCA patients and is increasingly used to assess for vessel involvement in PMR.

The timing of aortic damage evaluation was decided to address that the risk of aortic damage appears to be present from the time of diagnosis (39, 74, 78), with the cumulative incidence rising almost linearly over time (46) and to minimize death as a competing risk. Finally, it was considered feasible, to plan a 2-year follow-up imaging visit within the time frame of routine rheumatology care, minimizing loss to follow-up. Nevertheless, the timing also implies a risk of missing damage that is not yet detectable.

In September 2023, DANIVAS held its first annual DANIVAS research symposium to officially launch the DANIVAS cohort study to a broader audience of researchers and clinicians of the Danish rheumatology community and several rheumatology departments nationwide have committed to being part of the DANIVAS study. In November 2023, the first GCA and PMR patients were enrolled in the DANIVAS study. Within the next year, more Danish centers will be enrolled. On a longer perspective, the DANIVAS study is designed to improve the care and outcomes for patients with GCA and PMR.

## Ethics statement

The study has been conducted in full conformance with the principles of the Declaration of Helsinki. The Central Denmark Region Committees on Health Research Ethics (reference number 1–10–72-174-22) have approved the DANIVAS study protocol. The DANIVAS study is registered in the Danish Central Region internal list of research projects (reference number 1–16–02-470-22). All patients included in the study gave their written informed consent.

## Author contributions

BN: Conceptualization, Funding acquisition, Investigation, Methodology, Project administration, Writing – original draft, Writing – review & editing. SK: Conceptualization, Investigation, Methodology, Project administration, Writing – review & editing, Validation. AD: Investigation, Methodology, Project administration, Writing – review & editing. LT: Conceptualization, Investigation, Methodology, Project administration, Writing – review & editing. LD: Conceptualization, Investigation, Methodology, Project administration, Writing – review & editing. AC: Conceptualization, Investigation, Methodology, Project administration, Writing – review & editing. MH: Conceptualization, Investigation, Methodology, Project administration, Writing – review & editing. PH: Conceptualization, Investigation, Methodology, Project administration, Writing – review & editing. TE: Conceptualization, Investigation, Methodology, Project administration, Writing – review & editing. E-MH: Conceptualization, Funding acquisition, Investigation, Methodology, Project administration, Writing – review & editing. SC: Investigation, Methodology, Project administration, Writing – review & editing, Conceptualization. KK: Funding acquisition, Investigation, Methodology, Project administration, Writing – review & editing, Conceptualization.
