# Correction: The DANIsh VASculitis cohort study: protocol for a national multicenter prospective study including incident and prevalent patients with giant cell arteritis and polymyalgia rheumatica

**DOI:** 10.3389/fmed.2026.1833528

**Published:** 2026-03-31

**Authors:** Berit D. Nielsen, Salome Kristensen, Agnete Donskov, Lene Terslev, Lene Wohlfahrt Dreyer, Ada Colic, Merete Lund Hetland, Pil Højgaard, Torkell Ellingsen, Ellen-Margrethe Hauge, Stavros Chrysidis, Kresten K. Keller

**Affiliations:** 1 Department of Medicine, The Regional Hospital in Horsens, Horsens, Denmark; 2 Department of Rheumatology, Aarhus University Hospital, Aarhus, Denmark; 3 Department of Clinical Medicine, Aarhus University, Aarhus, Denmark; 4 Center of Rheumatic Research Aalborg (CERRA), Department of Rheumatology, Aalborg University Hospital, Aalborg, Denmark; 5 Department of Clinical Medicine, Aalborg University, Aalborg, Denmark; 6 DANBIO and Copenhagen Center for Arthritis Research (COPECARE), Center for Rheumatology and Spine Diseases, Centre for Head and Orthopedics, Rigshospitalet, Glostrup, Denmark; 7 Department of Clinical Medicine, Faculty of Health and Medical Sciences, University of Copenhagen, Copenhagen, Denmark; 8 Department of Rheumatology, Zealand University Hospital, Køge, Denmark; 9 Department of Medicine (2), Holbæk Hospital, Holbæk, Denmark; 10 Department of Rheumatology, Odense University Hospital, Odense, Denmark; 11 Department of Rheumatology, University Hospital of Southern Denmark, Esbjerg, Denmark

**Keywords:** giant cell arteritis, polymyalgia rheumatica, imaging, prognosis, cohort study (or longitudinal study), prospective observational study

The funder Health Research Foundation of the Central Denmark Region, [A3152] to Berit Dalsgaard Nielsen was erroneously omitted.

The original version of this article has been updated.

